# Non-RBM Mutations Impaired SARS-CoV-2 Spike Protein Regulated to the ACE2 Receptor Based on Molecular Dynamic Simulation

**DOI:** 10.3389/fmolb.2021.614443

**Published:** 2021-07-27

**Authors:** Yaoqiang Du, Hao Wang, Linjie Chen, Quan Fang, Biqin Zhang, Luxi Jiang, Zhaoyu Wu, Yexiaoqing Yang, Ying Zhou, Bingyu Chen, Jianxin Lyu, Zhen Wang

**Affiliations:** ^1^ Allergy Center, Department of Transfusion Medicine, Ministry of Education Key Laboratory of Laboratory Medicine, Zhejiang Provincial People’s Hospital, Affiliated People’s Hospital, Hangzhou Medical College, Hangzhou, China; ^2^ National Clinical Research Center for Child Health, National Children’s Regional Medical Center, Department of Clinical Laboratory, The Children’s Hospital, Zhejiang University Shcool of Medicine, Hangzhou, China; ^3^ School of Laboratory Medicine, Hangzhou Medical College, Hangzhou, China; ^4^ Department of Hematology, Zhejiang Provincial People’s Hospital, Affiliated People’s Hospital, Hangzhou Medical College, Hangzhou, China; ^5^ School of Laboratory Medicine and Life Sciences, Wenzhou Medical University, Wenzhou, China

**Keywords:** SARS-CoV-2, spike protein, non-RBM mutations, ACE2 receptor, molecular dynamic simulation

## Abstract

The emergence of novel coronavirus mutants is a main factor behind the deterioration of the epidemic situation. Further studies into the pathogenicity of these mutants are thus urgently needed. Binding of the spinous protein receptor binding domain (RBD) of SARS-CoV-2 to the angiotensin-converting enzyme 2 (ACE2) receptor was shown to initiate coronavirus entry into host cells and lead to their infection. The receptor-binding motif (RBM, 438–506) is a region that directly interacts with ACE2 receptor in the RBD and plays a crucial role in determining affinity. To unravel how mutations in the non-RBM regions impact the interaction between RBD and ACE2, we selected three non-RBM mutant systems (N354D, D364Y, and V367F) from the documented clinical cases, and the Q498A mutant system located in the RBM region served as the control. Molecular dynamics simulation was conducted on the mutant systems and the wild-type (WT) system, and verified experiments also performed. Non-RBM mutations have been shown not only to change conformation of the RBM region but also to significantly influence its hydrogen bonding and hydrophobic interactions. In particular, the D364Y and V367F systems showed a higher affinity for ACE2 owing to their electrostatic interactions and polar solvation energy changes. In addition, although the binding free energy at this point increased after the mutation of N354D, the conformation of the random coil (Pro384-Asp389) was looser than that of other systems, and the combined effect weakened the binding free energy between RBD and ACE2. Interestingly, we also found a random coil (Ala475-Gly485). This random coil is very sensitive to mutations, and both types of mutations increase the binding free energy of residues in this region. We found that the binding loop (Tyr495-Tyr505) in the RBD domain strongly binds to Lys353, an important residue of the ACE2 domain previously identified. The binding free energy of the non-RBM mutant group at the binding loop had positive and negative changes, and these changes were more obvious than that of the Q498A system. The results of this study elucidate the effect of non-RBM mutation on ACE2-RBD binding, and provide new insights for SARS-CoV-2 mutation research.

## Introduction

Several cases of unexplained pneumonia occurred in Wuhan, China, at the end of 2019 (Wang et al., 2020a). Patients’ clinical symptoms were related to infectious atypical pneumonia (SARS-CoV) and Middle East respiratory syndrome (MERS-CoV), such as fever, cough, and difficulty breathing (Huang et al., 2020). After investigation, it was found that the first case of pneumonia originated from a seafood and farmer’s market in Wuhan. Whole-genome sequencing revealed that the pathogen causing pneumonia was a new type of coronavirus. The virus was named 2019-nCoV by the World Health Organization and was subsequently renamed SARS-CoV-2 by the International Commission for Classification of Viruses (Tan et al., 2020). The novel virus has become a global public health event, and many countries have been deeply affected. More than 176 million patients have been infected, and there have been about 3.8 million deaths worldwide until June 2021.

Immediately after the virus broke out, it was sequenced on January 9, 2020, and the sequencing data were posted on the Internet afterwards (Gralinski and Menachery, 2020). Coronaviruses are 26,000 to 32,000 bases in length and are single-stranded positive-stranded RNA (Su et al., 2016). They mainly infect the respiratory tract and digestive tract. According to their genome characteristics, they can be divided into α-coronavirus, β-coronavirus, γ-coronavirus, and δ-coronavirus (Li, 2016). There are six types of coronaviruses that can cause human infection, two of which belong to α-coronavirus, and four belong to β-coronavirus (Tang et al., 2015), and the remaining virus types cannot infect humans. The most aggressive coronaviruses are SARS-CoV and MERS-CoV (belonging to β-coronavirus), as both of these viruses have caused global public health events. These two viruses are believed to have been transmitted from bats to civet cats or camels and, eventually, to humans (Guan et al., 2003; Azhar et al., 2014; Cui et al., 2019). SARS-COV-2 has a Spike protein has 93.1% homology with RaTG13 (bat-like coronavirus), but it has less than 80% homology with other SARS-CoV (Zhou et al., 2020).

To infect the human body, the virus must first bind to the corresponding receptor. For example, angiotensin-converting enzyme 2 (ACE2), CD209L, and dipeptidyl peptidase 4 (DPP4) were the main receptors for the SARS-CoV outbreak in 2003 and MERS-CoV in 2012 (Wu et al., 2020). The receptor of SARS-CoV-2 is also ACE2 (Wang et al., 2020b). After the virus enters the human body, the receptor binding domain (RBD) on the virus Spike protein should first bind to the receptor of the cell before it can fuse with the cell membrane and complete the infection. The Spike protein of SARS-CoV-2 is a structural protein encoded by the end of the viral genome (Wu et al., 2020), when it binds to cell-related receptors, it will be broken down into two subunits, S1 and S2, of which the S1 subunit will directly bind to the receptor (Wang et al., 2020b). Structural changes in RBD may lead to enhanced or weakened binding of the virus to the receptor (Lan et al., 2020) and then affect the probability of virus infection. Here we found a mutant of SARS-CoV-2, which will cause changes in the amino acid sequence of the SARS-CoV-2 RBD region, but the consequences of this change are not yet clear. We used molecular dynamics (MD) simulation methods to describe and compare various atomic forces in wild-type (WT) and mutant SARS-CoV-2 RBD and try to have a more detailed understanding of the binding energy changes caused by mutations, which will help promote new targeted therapies against this emerging pathogen.

As for the relationship between ACE2 and the RBD region, the role of the receptor-binding motif (RBM) is self-evident. The amino acid changes in other regions could also have an impact on the overall population. A previous study showed that the D614G mutation in the Spike protein could increase viral infectivity (Hu et al., 2020). The G614 mutation can enhance the ability of protease to cut the Spike protein, thus promoting the virus to be more infectious. We constructed three mutations on the basis of the RBD information found in actual cases (Ou et al., 2021) and selected the important 498 residues in RBM for the alanine mutation as the control group. We aim to explore the impact of mutations in different regions of RBD on its binding effect and pay attention to the changes in the RBM region.

## Results and Discussion

### Predicting Mutation Effects on Protein–Protein Interactions

Protein–protein interactions (PPIs) play an important role in various biological processes that include cell regulation and signal transduction. Various studies have shown that many disease-related amino acid mutations are located at the protein–protein interface, thereby affecting PPIs by changing the binding affinity or specificity. SAAMBE-3D is a newly developed machine learning algorithm used to predict the impact of single amino acid mutations on PPIs (Pahari et al., 2020). Using the PDB structure of 6LZG as the input file, the binding free energy change caused by the mutation and the prediction of whether the mutation disturbs PPIs were obtained. According to the analysis of the results in Table 1, the binding energy of N354D and D364Y were reduced, and the entire PPI networks were destroyed. On the contrary, the binding energy of the Q498A mutation located in the RBM region was also reduced, but the PPI network was not destroyed. Together, these results using a machine learning algorithm revealed that residue mutations that are not present in the RBM region may have a greater effect on the complex, a finding that we found particularly interesting. Considering the limited amount of raw data and the particularity of the genome, we then used dynamic simulation to unravel detailed outcomes.

**TABLE 1:** The predicted effect of single amino acid mutation of PPIs.

System	N354D	D364Y	V367F	Q498A
ΔG Prediction^a^	0.59	0.59	−0.42	2.93
Effect	Disruptive	Disruptive	Nondisruptive	Nondisruptive

a Prediction of changed binding free energy (kJ/mol) caused by the mutation.

### Structural Flexibility and Stability of the Simulation Systems

**FIGURE 1:**
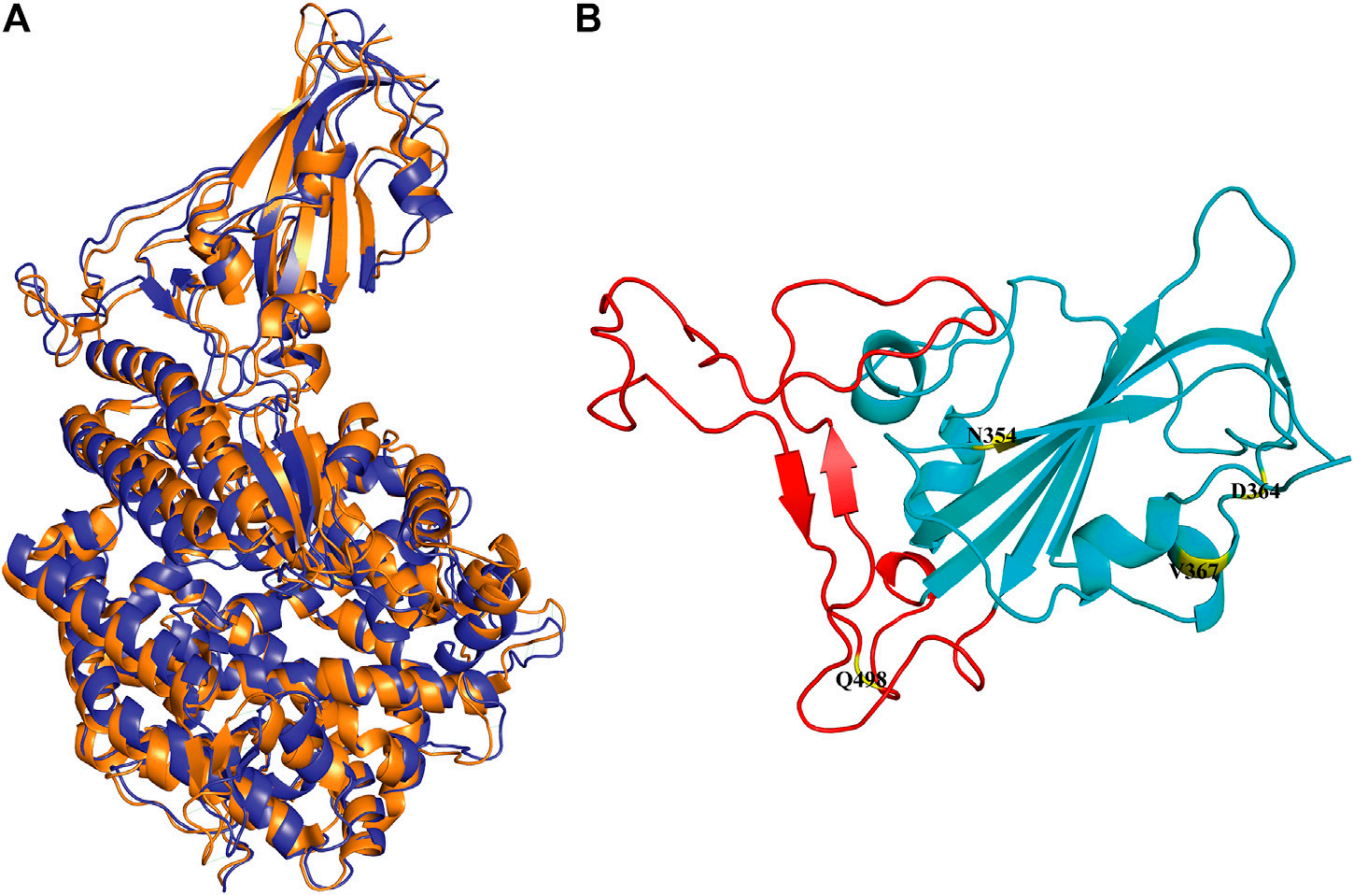
Conformation of WT RBD/hACE2 and position of four mutations in the RBD domain. **(A)** The crystal is illustrated in orange, and the representative structure after simulation is in blue. **(B)** The four mutation sites are shown in yellow, and the range of red fragments represents RBM.

In the simulation process, the superposition of representative structures with the crystal shows that they are very similar (Figure 1A), which indicates that the simulation was performed under ideal conditions. The non-RBM mutations are found structurally far away from the RBM region (Figure 1B), conducting kinetic simulation could thus provide insights into their interactions.

**FIGURE 2:**
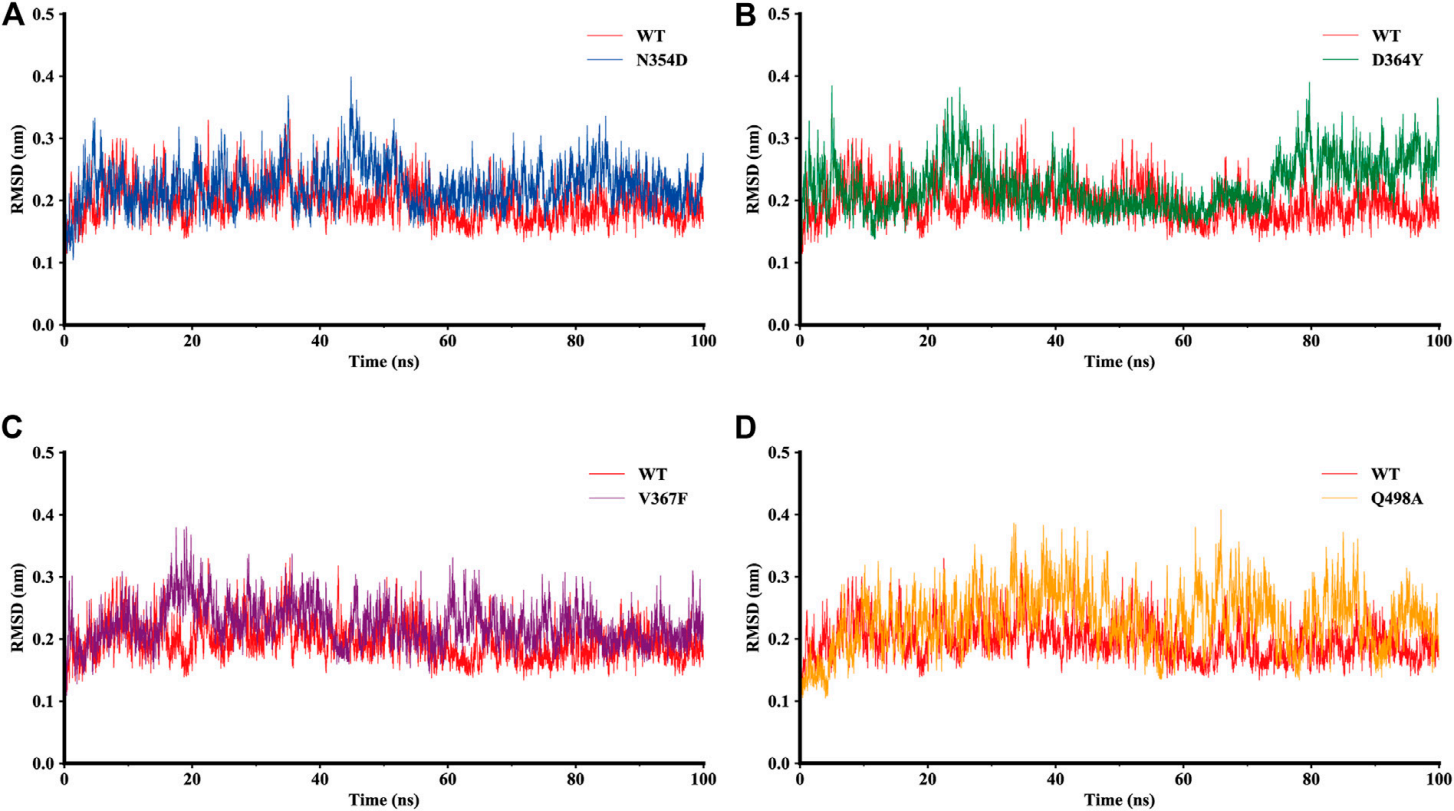
RMSD of backbone atoms relative to the initial structure during the MD trajectories. **(A)** RMSD values in the WT system (red) and N354D system (blue). **(B)** WT system and D364Y system (green). **(C)** WT system and V367F system (purple). **(D)** WT system and Q498A system (orange).

The dynamic behavior of the WT system and the mutant system were analyzed by 100-ns MD simulations. The stability of the WT and mutant systems was determined using the root-mean-square deviation (RMSD) of the backbone atoms relative to the initial structure (Figure 2). It can be seen from the figure that the conformations of the five complexes reached equilibrium 10 ns after the start of the simulation, with no obvious fluctuations. The RMSD values of N354D, D364Y, and V367F in the non-RBM group all approximated 0.22 nm, which is slightly higher than the 0.19 nm value observed in the WT system (Figures 2A–C). The RMSD value of Q498A in the RBM group was 0.23 nm, which was higher than for the other four systems (Figure 2D). This indicates that the stability of mutant systems is lower than that of the WT system and that the Q498A system located in the RBM region is the most unstable.

**FIGURE 3:**
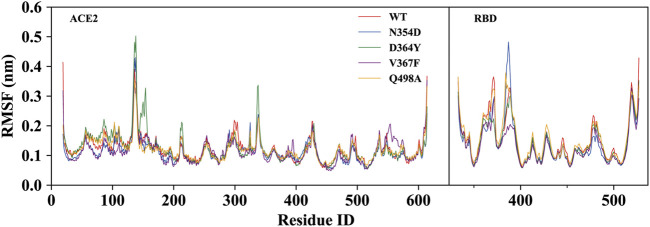
RMSF of backbone atoms relative to the initial structure during the MD trajectories. RMSF values of ACE2 and RBD in the WT system (red), N354D system (blue), D364Y system (green), V367F system (blue) and Q498A system (orange).

To investigate the detailed residual atomic fluctuations, the Gromacs was applied to compute the root-mean-square fluctuation (RMSF) of the backbone atoms versus residue ID for all systems (Figure 3). It is generally believed that the fluctuation of the residue was high if the RMSF value was equal to or greater than 0.23 nm. Comparing the fluctuations of the RMSF value between different systems, we found an area with large fluctuations, which is a random coil (Pro384-Asp389) between β2 and β3 in the RBD domain. The N354D system displayed the highest flexibility in this segment, whereas the V367F system, also from the non-RBM group, had the lowest flexibility. Although this segment was not the key area of the combination, its influence cannot be ignored. Coincidentally, the random coils in the RBM region (Ala475-Gly485) of all mutant systems had high RMSF values, suggesting that this region was sensitive to both types of mutations. In addition, the random coil (Asn134-Glu140) between α1 and α2 in the ACE2 domain of the D364Y system fluctuated greatly, and the RMSF value was 0.5 nm, compared to 0.4 nm for the WT system. Therefore, we speculate that D364Y not only changes the conformation of the RBD domain but also affects the binding state of the complex. In order to understand the specific changes of the random coil described, we further analyzed secondary structures over time.

### Secondary Structure Analysis of Fluctuation Regions

**FIGURE 4:**
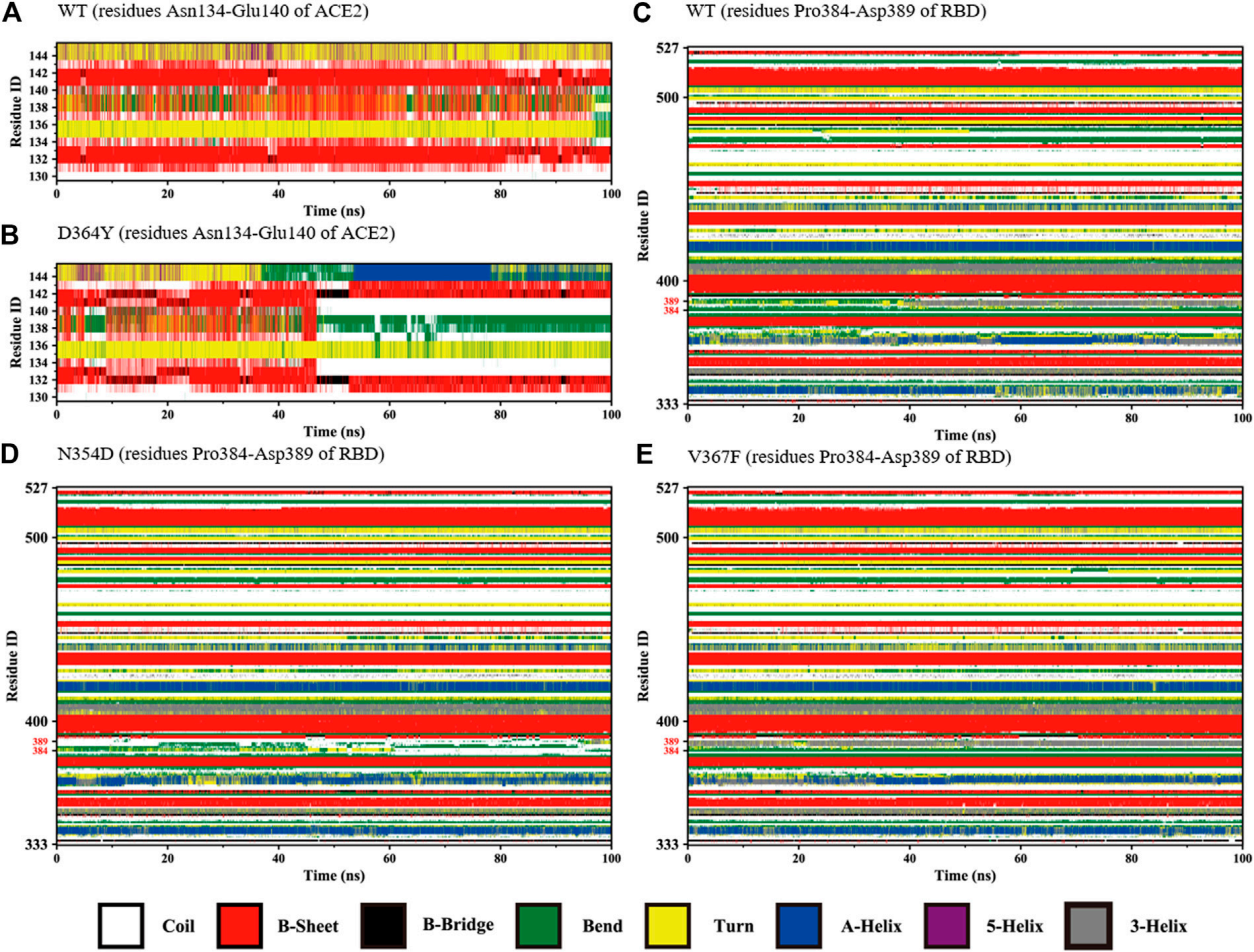
Secondary structures of the regions (residues Asn134-Glu140 of ACE2 and Pro384-Asp389 of RBD) during the simulation. **(A)** Residues Asn134-Glu140 of ACE2 in the WT system. **(B)** Residues Asn134-Glu140 of ACE2 in the D364Y system. **(C)** Residues Pro384-Asp389 of RBD in the WT system. **(D)** Residues Pro384-Asp389 of RBD in the N354D system. **(E)** Residues Pro384-Asp389 of RBD in the V367F system.

The changes with time of the secondary structures were obtained for each amino acid using DSSP plugin analysis in Gromacs. We compared the fluctuations of random coils (Asn134-Glu140) in the ACE2 domain of the D364Y and WT systems. At the beginning of the simulation, the secondary structures of the two systems in this region was found to fluctuate between β-sheets and bends (Figures 4A,B). As the simulation progressed, the secondary structure of the D364Y system changed from β-sheet to coil after 47 ns(Figure 4B). This change enhanced the flexibility of the region while affecting the upstream binding of α1 to RBM. By comparing the proportion of secondary structures in the two domains of the complex, we found that the helical ratio of the RBD domain was much smaller than that for the ACE2 domain, which indicated that the conformation of the virus was unstable and prone to mutation. The random coil Pro384-Asp389 in the WT system is a mixture of 3-helix, bend, and turn (Figure 4C), but the 3-helix in the N354D system is almost entirely replaced by coil (Figure 4D). This indicates that the N354D mutation increases the flexibility of this region and thus affects the spatial position of adjacent secondary structures. In contrast, the V367F system was almost entirely 3-helix during the whole simulation (Figure 4E), which indicated that the conformation of the random coil Pro384-Asp389 of the V367F system was very stable. In addition, comparison with the WT system revealed that the secondary conformation of both the D364Y and Q498A systems did not change significantly in this region (Supplementary Figures S1A,B).

**FIGURE 5:**
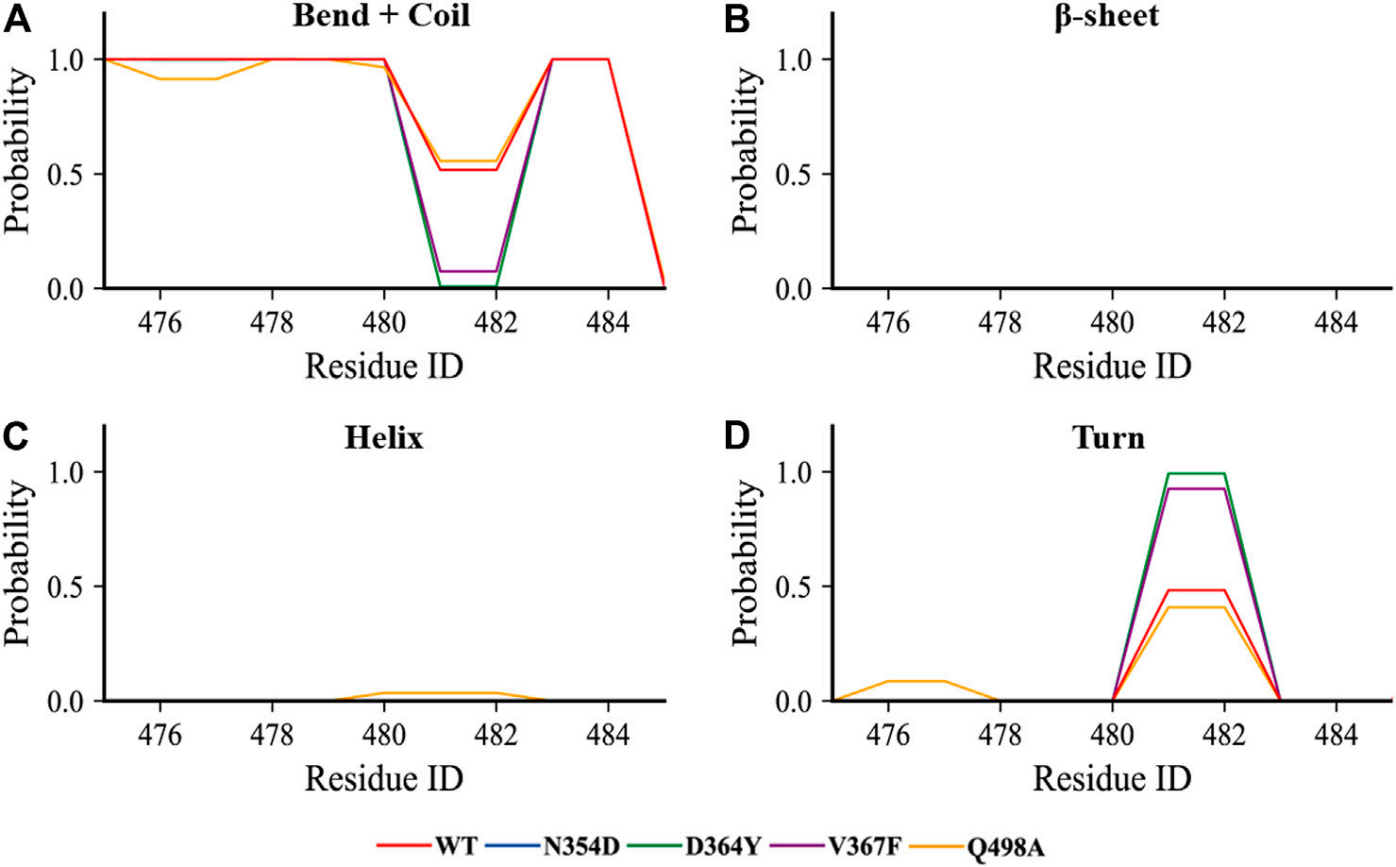
The content of various secondary structures in the region (Ala475-Gly485) in the RBD of each system. **(A**–**D)** The proportions of the four secondary structures in this region are bend + coil, β-sheet, helix, and turn.

Considering that the RMSF values of the random coil (Ala475-Gly485) were slightly different, we used the DSSP tool to calculate the proportion of secondary structures in this region. All the coils in the non-RBM group became turn (Figure 5A), while the Q498A and WT systems showed a similar trend, they were converted from coil to turn in less than half of the simulation frames (Figure 5D). These results indicate that this region is more sensitive to non-RBM mutations, which decrease the flexibility of the region.

### Binding Free Energy and Decomposition Analyses of Mutant and WT Complexes

The binding free energy was calculated as the sum of gas phase energy (ΔE_vdw_ + ΔE_ele_), solvation free energy (ΔG_SA_ + ΔG_PB_), and entropy (−TΔS). Recent studies have shown that the interaction entropy (IE) method provides more reliable predictions for the free energy of protein–ligand and protein–protein binding and entropy contribution of hot residue interactions. The results showed that the binding free energy of the N354D system was −52.085 kJ/mol, lower than that of the WT system −85.611 kJ/mol. The binding free energy of the D364Y, V367F and Q498A was higher than that of the WT system, which was −298.563, −200.852, and −222.705 kJ/mol, respectively (Table 2).

**TABLE 2:** Binding free energy components for the ACE2-RBD complex by using the MM-PB/SA method.

Component (kJ/mol)	WT	N354D	D364Y	V367F	Q498A
$\Delta E_{\mathrm{vdw}}$	−383.448	−375.295	−392.392	−406.108	−382.819
$\Delta E_{\mathrm{ele}}$	−811.617	−587.415	−873.228	−707.774	−686.019
$\Delta G_{\mathrm{polar}}$	1086.042	861.460	920.047	871.004	795.021
$\Delta G_{\mathrm{nopolar}}$	−47.215	−47.084	−47.618	−48.289	−47.176
ΔH ^a^	−156.238	−148.335	−393.191	−291.167	−320.993
−TΔS	70.627	96.277	94.628	90.315	98.288
$\Delta G_{\mathrm{bind}}\;$ **^b^**	−85.611	−52.085	−298.563	−200.852	−222.705

a $\Delta \text{H}=\Delta {\text{E}}_{\text{vdw}}+\Delta {\text{E}}_{\text{ele}}+\Delta {\text{G}}_{\text{polar}}+\Delta {\text{G}}_{\text{nopolar}}$.

b $\Delta {\text{G}}_{\text{bind}}=\Delta \text{H}-\text{T}\Delta \text{S}$.

By comparing the decomposition energies, we found that the main driving force in the binding process is the polar solvation energy, which is the difference between the electrostatic energy of solvent and vacuum. The contributions of the polar solvation energy of the WT, N354D, D364Y, V367F, and Q498A systems are 1,086.042, 861.460, 920.047, 871.004, and 795.021 kJ/mol, respectively. The polar solvation energy of all mutation systems were decreased, and the Q498A system changed the most, which may be related to the hydrophobicity of alanine. The polar solvation energy of all mutant systems is lower than that of WT system, which indicated that the conformational change reduces the solubility of the RBD domain in the polar solution. In these five systems, the relatively small non-polar solvation energy indicated that the packing of the cavity region is quite closed. Similar to the non-polar solvation energy, the van der Waals force in the five systems were not different from each other. The electrostatic interaction of the D364Y system is 62 kJ/mol, which is higher than that of the WT system, whereas the electrostatic interaction of the N354D system is much smaller than that of the other four systems. As a result, the electrostatic interaction was the main factor leading to the change in the binding free energy of the N354D and D364Y systems. For the V367F and Q498A systems, although the electrostatic interaction and polar solvation energy were reduced, the polar solvation energy decreased more dramatically and the binding free energy of these two systems (V367F and Q498A) was higher than that of the WT system. The interaction entropy was determined by the floating of the binding energy in the gas phase, and the WT system has the lowest entropy value, which is similar to the previous RMSD numerical trend. It is important to emphasize here that, in the Amber force field, electrostatic interactions were described by fixed-point charge interactions without considering the electrostatic polarization effect of protein. This may be the reason why the values of our combined free energy are not completely consistent with the experimental results.

In order to clarify the specific impact of mutations on the binding affinity of the system, we decomposed the binding free energy to each residue (Supplementary Table S1). In the RBD domains of all systems, the top amino acids that contributed to binding free energy were Lys and Arg, and their electrostatic energy contributions were much higher than those of other amino acids. The electrostatic interaction of these basic amino acids was the main reason for the stable combination of ACE2 and the RBD domain. For the N354D system, the polarity of the mutation point did not change, but an uncharged amino acid changed to a charged amino acid. The most obvious observation is that the electrostatic interaction at the 354 mutation site was significantly reduced, whereas the solvation free energy is only slightly reduced. Because of the mutation at site 354, the binding free energy contribution at this point changed from 0.52 to 80.07 kJ/mol. This should be the main reason for the significant reduction of electrostatic interaction in the N354D system. The mutation of charged Asp to uncharged Tyr in the D364Y system modified the electrostatic interaction at site 364 from 71.99 to 0.01 kJ/mol. In addition, the binding free energy of the seven amino acids contained in the random coil (Asn134-Glu140) of the system was increased, and the total binding energy was reduced to −16.51 kJ/mol (Supplementary Table S1), compared with −11.35 kJ/mol for the WT system. The polarity and electrification of the V367F system residue did not change, but the binding free energy was greater than that of the WT system. This may be related to the residue Tyr449 in the RBM region, and its polar solvation energy decreased to 13 kJ/mol. Similar to that of Tyr449, the polar solvation energy of other residues in the RBM region was also improved to different degrees. Because the mutated alanine in the Q498A system is a hydrophobic amino acid, the original strong polar solvation energy of this site is weakened. At the same time, the mutation of the Q498A system caused the loss of glutamine hydrogen donor, which also led to the loss of hydrogen bonds. The superimposed effect of electrostatic interaction and polar solvation energy resulted in an increase in the binding energy contribution of residue 498 by 7 kJ/mol. In addition, the random coil (Ala475-Gly485) mentioned above is an interesting structure considering that its binding free energy increased whether the mutation was located in the RBM region or not.

### Hydrogen Bonds and Bonding Interfaces in RBM

The hydrogen bonds between the RBD and ACE2 were extracted using the Gromacs program with default criteria (D–A distance cutoff = 0.35 nm and H–D–A angle cutoff = 30°, where D, A and H are the donor atom, acceptor atom, and hydrogen atom linked to the donor atom, respectively), and we only retained the hydrogen bond pairs whose trajectory ratio were more than 20% (Supplementary Table S2). All the mutant systems had fewer hydrogen bonds than had the WT system; in particular, the V367F system only produced 14 hydrogen bonds. Lys417 is the only residue that was not located in the RBM region, interacting instead with Asp30 in the ACE2 domain to form hydrogen bonds and salt bridges. Moreover, the hydrogen bond of 417LYS-30ASP was found to account for more than 70% of all hydrogen bonds in all the investigated systems, indicating that this hydrogen bond is very important for the stability of the complex. Compared with the WT system, both the non-RBM group and the Q498A system lacked six sets of hydrogen bonds [34HIS (HE2)-494SER (O), 487ASN (D21)-24GLN (O), 498GLN (E21)-38ASP (OD1), 500THR (HG1)-355ASP (OD1), 478THR (HG1)-24GLN (OE1), and 24GLN (E21)-487ASN (OD1)]. The breaking of these hydrogen bonds has a certain relationship with the aforementioned changes in the secondary structure of the RBM region. Many studies have shown that hydrogen bonds play a crucial role in the structural stability of proteins, and the disappearance of hydrogen bonds can change the biological activity of protein (Joh et al., 2008). A new 493GLN (E21)-34HIS (O) hydrogen bond was generated in both the N354D and V367F systems after excluding the hydrogen bond contained in the WT system. The D364Y and Q498A systems lost the 353LYS (HZ1)-496GLY (O) hydrogen bond, but formed three new hydrogen bonds: 500THR (HG1)-355ASP (OD2), 353LYS (HZ1)-495TYR (O), and 83TYR (HH)-487ASN (ND2). Although mutations in the Q498A system resulted in the disappearance of all associated hydrogen bonds, the formation of new hydrogen bonds near position 498 kept the entire RBM region stable.

A residue is considered part of the interface if one of its atoms is within 0.4 nm from any atom of the other partner in at least 30% of the 10,000 MD simulation frames (10 ps as an interval). Taking this as the standard, we also analyzed the occupancy rate of the combined interface in the simulation process (Supplementary Table S3). We found that there is a binding loop (Tyr495-Tyr505) in the RBM region that is the main component of the binding interface. Then we visualize the binding interface of the binding loop in order to understand the specific details.

### Hydrogen Bonds and Hydrophobicity of the Binding Loop

**FIGURE 6:**
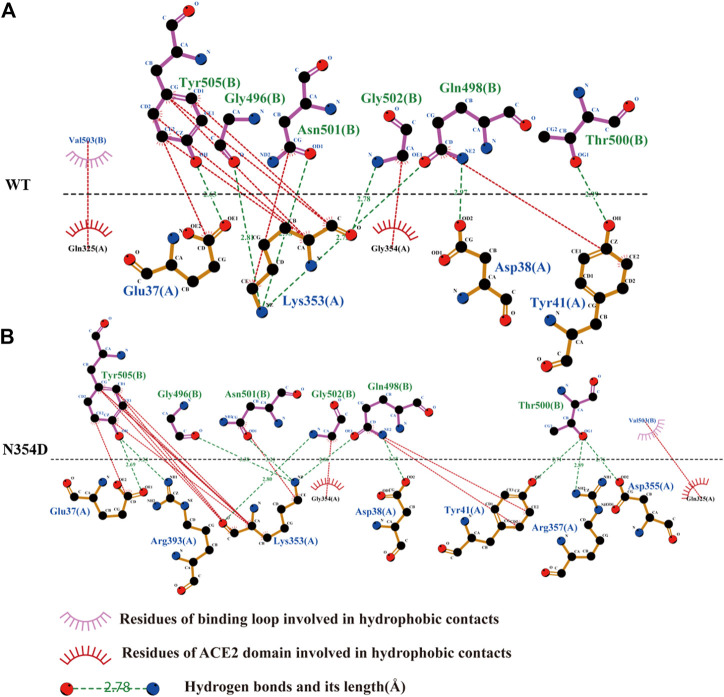
Network of interactions associated with binding loop in RBD derived from Ligplot+ software analysis. **(A)** Analysis of hydrogen bond and hydrophobic interaction of binding interface in the WT system. **(B)** N354D system.

We obtained the representative structure of each system through the clustering method, and then used Ligplot+ to display the information of the complex binding interface, which helps us understand the details of the binding loop (Tyr495-Tyr505) (Laskowski and Swindells, 2011). In all systems, Lys353 of the ACE2 domain has the most hydrogen bond combinations at the binding loop, indicating that it is a key residue in the binding interface. We speculate that this may be related to the strong hydrophobic interaction between Lys353 and Tyr505, which makes the binding loop and Lys353 tightly bound together (Figure 6A). This is also consistent with previous findings that mutation in Lys353 caused a significant reduction in binding free energy (Lupala et al., 2020).

The binding loop of the N354D system (Figure 6B) of the RBM group generates the most hydrogen bonds and hydrophobic interactions, which is related to the changes in the adjacent random coil (Ala475-Gly485). In the D364Y system, the hydrogen bond between Lys353 and the binding loop of Tyr505 was disrupted, significantly increasing the distance of their hydrophobic interaction (Supplementary Figure S2A). Although no hydrogen bond was formed at site 498 in the binding interface of both the V367F (Supplementary Figure S2B) and Q498A (Supplementary Figure S2C) systems, the hydrophobic interaction at this site was found to increase. The free energies of the binding loop in WT, N354D, D364Y, V367F and Q498A were of 8.30, 5.30, 13.51, 6.38, and 8.11 kJ/mol, respectively. These findings indicate that mutations in the non-RBM group have a greater effect on the binding loop, with these mutations either increasing or decreasing the binding free energy.

### Principal Component Analysis of the RBM Region

**FIGURE 7:**
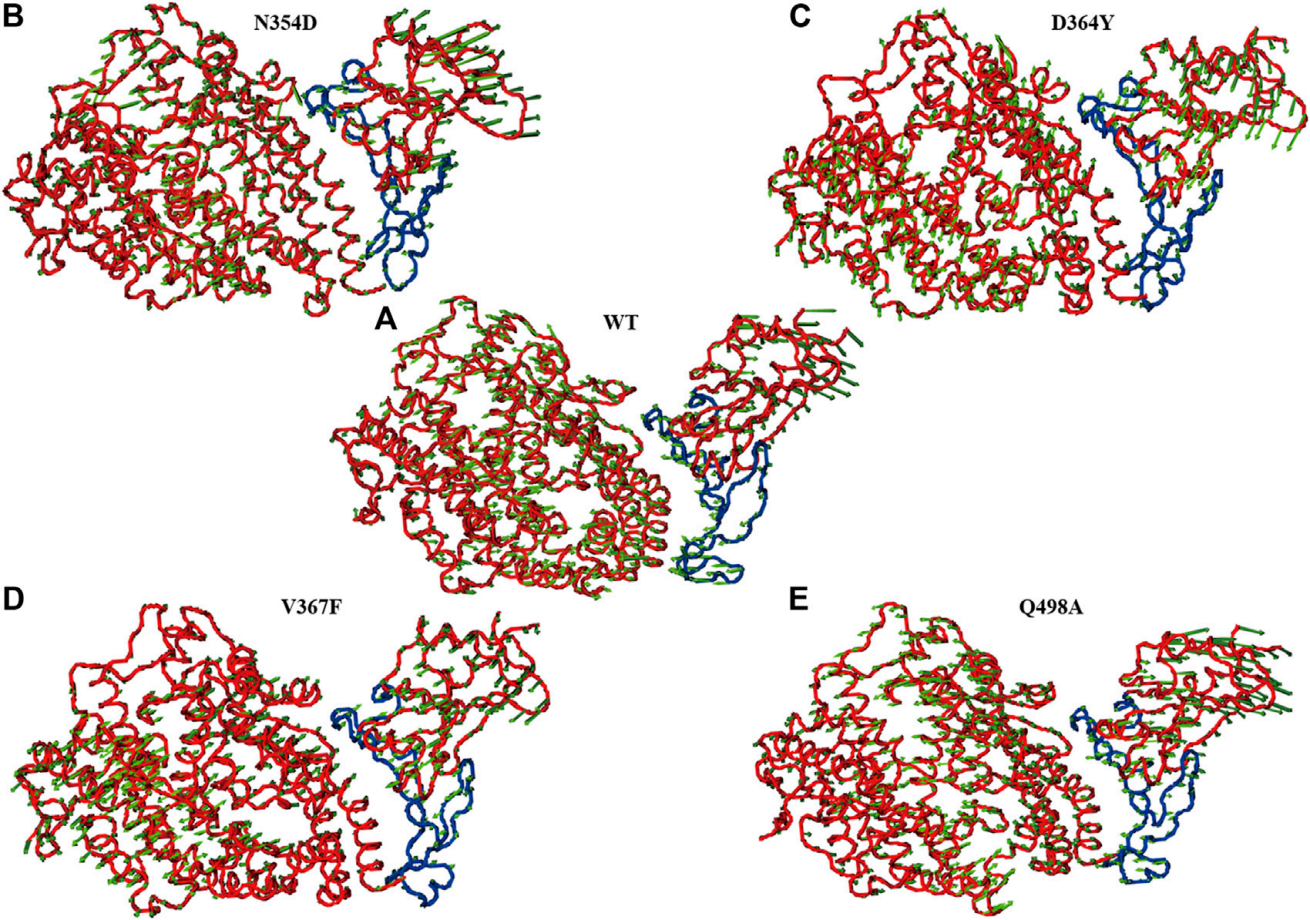
Principal component analysis of each system. The arrow (green) plot of the first (PC1) motion modes for the RBM (blue) region in the WT system **(A)**, N354D system **(B)**, D364Y system **(C)**, V367F system **(D)**, and Q498A system **(E)**.

The first principal component (PC1) could reflect large-amplitude motions of the protein C-alpha conformations, as illustrated in the principal component analysis (PCA) for the WT and mutant systems (Figure 7). The PCA explained how the mutations affect the motions. The direction of the motion is indicated by the direction of the arrow, and the magnitude of the motion is expressed by the length of the arrow. The RBM region (marked blue) in the N354D system has much less motion amplitude than has the WT system (Figures 7A,B), and the motion direction is disordered. The motion direction of the ACE2 domain at the interface is also irregular, which is the main reason for the decrease of binding energy of the system. In sharp contrast, the interface of ACE2 and RBD in the D364Y system has the same motion trend (Figure 7C), which makes the binding state between them very stable. The movement trend of the RBM region in the V367F system deviated from some angles (Figure 7D), which may be related to the overall decrease of the polar solvation energy in the RBM region. In the RBM region of the Q498A system (Figure 7E), the movement trend of most residues is similar to that of the WT system, but the movement trend of residues near the alanine mutation point is chaotic, which may be related to the rearrangement of water molecules around the mutation point. In summary, the motion of the RBM region in the complex is closely related to the binding free energy, and the motion direction and amplitude indirectly indicate the binding effect of ACE2 and RBD.

### Cell–Cell Fusion Assay Analysis of the Binding Between Mutated Spike and ACE2

**FIGURE 8:**
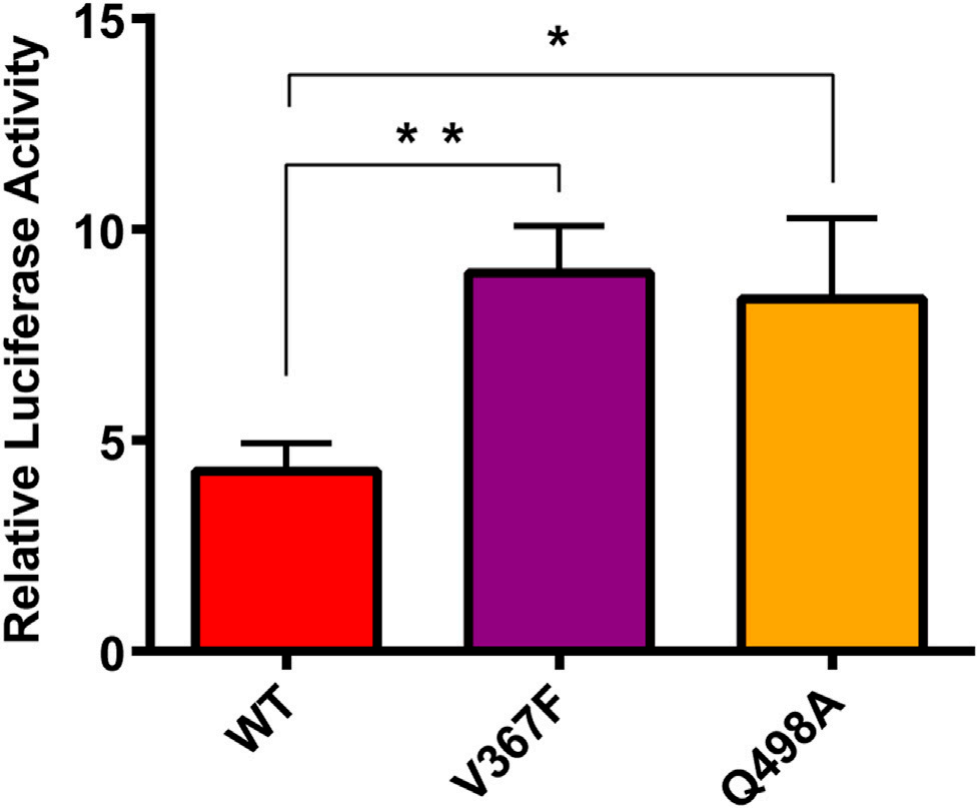
Cell-cell fusion assay analysis of the binding between mutated spike and ACE2. HEK293T cells expressing with pcDNA3.1-spike/mutant spike were co-incubated with cells expressing ACE2. After 48 h, Cells were lysis and the luciferase activity were detected. One-way ANOVA and Sidak’s multiple comparisons test were performed by GraphPad PRISM 6. *p < 0.05; **p < 0.01.

The Spike protein mutants N354D, D364Y, and especially V367F, which has been detected at high density (Gong et al., 2020; Song et al., 2020; Zhao et al., 2020), were detected in the early breakout area. We conducted cytological experiments to confirm the functional outcomes of V367F mutation on the binding of Spike protein with ACE2. WT and Q498A mutant were used as controls (Figure 8). A cell–cell fusion assay was used to mimic the SARS-CoV-2 virus spreading among ACE2-expressing cells (Kruglova et al., 2021; Zeng et al., 2021). This result showed that compared with the WT (RLU Ratio = 4.27 ± 0.54), the V367F mutation enhanced luciferase activity (RLU Ratio = 8.97 ± 0.91). This indicates that the affinity between Spike protein and human ACE2 is enhanced after V367F mutation. The Q498A mutation also increased luciferase activity (RLU Ratio = 8.36 ± 0.61), but it was slightly lower than the V367F mutation. This is slightly different from the simulation results, which may be due to the influence of other sequences in Spike protein that do not include RBD region. Consistent with the result of our cell-cell fusion assay, recently, a study also found the enhanced affinity and infectivity of the V367F Spike mutant based on ELISA, SPR and the pseudovirus entry assay (Ou et al., 2021), implying the important role of V367F mutant in this epidemic strain.

## Conclusion

Whether the mutation is located in the RBM region or not, it will change the overall stability of the complex. The secondary structure of the random coil Pro384-Asp389 of the N354D system and the V367F system has changed, which may indirectly affect the conformation of the RBM region. We also found a region (Ala475-Gly485) with obvious contrast, the secondary structure of the non-RBM group in this region was mostly changed from bend + coil to turn, whereas the Q498A system remained unchanged. Except for that of the N354D system, the binding free energy of the other three mutation systems is enhanced, and the electrostatic energy provides the greatest contribution. The chargeability of the base acid before and after the mutation in the N354D system and the D364Y system is different, which makes the electrostatic energy of the mutation site greatly changed, so the energy contribution change at this site is the main reason for the overall binding free energy change.

However, the V367F system of the non-RBM group is similar to the Q498A system. The decrease in polar solvation energy of most residues in the RBM region is the main reason for the increase in the overall binding free energy. We found that there are six extremely sensitive hydrogen bonds in the RBM region, which are broken in all mutation systems. However, the key hydrogen bonds that maintain the stability of the system have a high occupancy rate in the simulation process, such as the hydrogen bond 417LYS-30ASP located outside the RBM region. In addition, through the analysis of the binding interface of the binding loop, it can be seen that Lys353 of the ACE2 domain is tightly wrapped inside the binding ring and forms a strong hydrogen bond and hydrophobic effect. Therefore, we believe that Lys353 is an important residue in the binding interface, which is also consistent with the results of previous study. In the three-dimensional motion diagram of PCA, the movement direction of the RMB region in the N354D system is disordered and the motion amplitude is very low. The D364Y, V367F, and Q498A systems show little difference in the movement of the RBM region compared with the WT system. A cell–cell fusion assay was used to mimic the SARS-CoV-2 virus spreading among ACE2-expressing cells, thus the V367F and Q498A mutant displayed a significantly increased luciferase activity. In summary, this study reports the details of the changes in the binding of SARS-CoV-2 Spike protein RBD to the human ACE2 receptor by mutations in the non-RBM region. The non-RBM mutation will affect the secondary structure, hydrogen bonding, hydrophobic interaction and binding free energy, etc., and most of these effects act on the RBM region.

## Materials and Methods

### Preparation of the Structures

The SARS-CoV-2-RBD/hACE2 complex was obtained from the RCSB website named 6LZG (https://www.rcsb.org/) (Wang et al., 2020). The complex structure was solved at 0.25 nm resolution with a SARS-CoV-2-RBD binding to a single hACE2 molecule in the asymmetric unit. For hACE2, clear electron densities could be traced for 596 residues from S19 to A614 of the N-terminal peptidase domain, as well as glycans N-linked to residues 53, 90, and 322. In the complex structure, the SARS-CoV-2-RBD contains 195 consecutive density-traceable residues, spanning T333 to P527, together with N-linked glycosylation at N343.

On the basis of the WT structure, the starting structures of mutants N354D, D364Y, V367F, and Q498A were generated by the PyMOL software (Mooers and Brown, 2021). Five initial structures were prepared for the subsequent study. All missing hydrogen atoms were added using the pdb2gmx module in the Gromacs package (Van Der Spoel et al., 2005). The AMBER99SB-ILDN force field in Gromacs was applied to produce the parameters for the system (Lindorff-Larsen et al., 2010). Sodium ions were added to keep the whole system neutral. The system was solvated with water in a truncated octahedron box with a 1.5 nm distance around the solute, and the TIP3P model was used to describe the water (Van Der Spoel and van Maaren, 2006).

### Molecular Dynamics Simulations

MD simulations for all complexes were performed using the Gromacs package (Van Der Spoel et al., 2005). The particle mesh Ewald method was used to treat the long-range electrostatic interactions under periodic boundary conditions (Darden et al., 1993; Essmann et al., 1995). The short-range non-bonded interactions were calculated on the basis of a cutoff of 1 nm. The structures were initially fixed with a 1,000 kcal mol^−1^ nm^−2^ harmonic constraint, and both solvent and ions were energy minimized for 20,000 steps of the steepest descent method for each system. And then the systems were heated to 300 K in the NVT ensemble with velocity of 1 K ps^−1^. Then the restrain was gradually decreased within 1 ns from 1,000 to 0 kcal mol^−1^ nm^−1^. Finally, 100 ns MD simulations at a temperature of 300 K and a pressure of 1 atm were carried out without any restrain. The temperature was maintained at 300 K with the collision frequency 0.1 ps^−1^ using the Berendsen thermostat, and a constant isotropic press was maintained at 1 bar using the Berendsen barostat (Berendsen et al., 1984). All bonds involving hydrogen atoms were restricted using the SHAKE algorithm (Coleman et al., 1977; Ryckaert et al., 1977). A time step of 2 fs was used in all MD simulations.

### MM/PBSA Calculations

As one of the most widely used methods, MM/PBSA has always played a significant role on calculation of binding free energy. In the MM/PBSA approach, the binding free energyΔG_bind_ was calculated as follows:

$$\Delta G_{bind}=\Delta H-T\Delta S=<E_{pl}^{int}>+\Delta G_{sol}-T\Delta S$$ (1)

where ΔH represents enthalpy change and $<E_{pl}^{int}>$ represents the ensemble averaged protein–ligand interaction including electrostatic interaction and van der Waals (vdw) interaction. $\Delta G_{sol}$ and −TΔS represent the solvation free energy and contribution of entropy change, respectively. $\Delta G_{sol}$ can be divided into two parts:

$$\Delta G_{sol}=\Delta G_{pb}+\Delta G_{np}$$ (2)

where $\Delta G_{pb}$ and $\Delta G_{np}$ represent the polar and non-polar solvation free energy, respectively. $\Delta G_{pb}$ was calculated using the PB equation. The exterior and interior dielectric constants were set to 80 and 2, respectively. In the meantime, $\Delta G_{np}$ was calculated using the following equation:

$$\Delta G_{np}=\gamma \times SASA+\beta$$ (3)

where SASA represents solvent-accessible surface area, and it can be calculated using the g_mmpbsa program. The numerical values of *γ* and *β* are the standard values of 0.00542 kcal/(mol Å^2^) and 0.92 kcal/mol, respectively (Sanner et al., 1996). For each system, the average binding free energy was calculated for 500 snapshots extracted from the last 5 ns of the trajectories at 10-ps interval for the complex structure. The MM/PBSA energy decomposition was performed to address the contributions of each residue to the binding free energy.

### Interaction Entropy

In addition to the N mode, a new more rigorous and concise method, that is, the IE method, is employed to calculate entropy change. It can be defined as follows:

$$-T\Delta S=KT\ln<e^{\beta \Delta E_{pl}^{int}}>$$ (4)

where $\Delta E_{pl}^{int}$ represents the fluctuation of protein–ligand interaction energy ($E_{pl}^{int}$) around the average energy ($<E_{pl}^{int}>$). It can be calculated as follows:

$$\Delta E_{pl}^{int}\;=E_{pl}^{int}\;+\;<E_{pl}^{int}>$$ (5)

The protein–ligand interaction energy ($E_{pl}^{int}$) consists of electrostatic interaction and vdW interactions. The efficiency of this approach lies in the fact that the two averages $<E_{pl}^{int}>$ and $<e^{\beta \Delta E_{pl}^{int}}>$ can be calculated simply using the following equations:

$$<E_{pl}^{int}>\;=\;\frac{1}{N}\overset{N}{\underset{i}{\sum }}E_{pl}^{int}\left(t_{i}\right)$$ (6)

and

$$<E_{pl}^{int}>\;=\;\frac{1}{N}\overset{N}{\underset{i}{\sum }}e^{\beta \Delta E_{pl}^{int}\left(t_{i}\right)}$$ (7)

where *β* is 1*/KT.*

In the IE method, the residue decomposition of entropy change (Wang et al., 2017) is performed using the following equations:

$$-\text{T}\Delta S_{rl}=KT\ln<e^{\beta \Delta E_{rl}^{int}}>$$ (8)

where $\Delta E_{rl}^{int}$ represents the fluctuation of residue–ligand interaction energy ($E_{rl}^{int}$) around the average energy ($<E_{rl}^{int}>$).

### Clustering and Hydrogen Bonding

The cluster analysis of protein conformations was carried out using Gromos as the clustering algorithm and all-protein atom RMSD as the similarity metric. The cluster analysis performed as follows: count the number of neighbors using a cutoff value, take the structure with the largest number of neighbors with all its neighbors as a cluster, and eliminate it from the pool of clusters. Repeat for the remaining structures in the pool (Daura et al., 1999).

Hydrogen bonds were determined via the distance between the D and A heavy atoms using a cutoff value of 0.35 nm and the angle H–D–A using a cutoff value of 30° (Van Der Spoel et al., 2005).

### Principal Component Analysis

PCA is one of the most popular postdynamic techniques (Kurylowicz et al., 2010) that has been widely used to provide a better understanding of the dynamics of a biological system. It defines atomic displacement in a collective manner that transforms the original HD set of (possibly) correlated variables into a reduced set of uncorrelated variables—the principal components (PCs). The most significant fluctuation modes of a protein together with the motion of the system can be identified using PCA in terms of planarity of motion (eigenvectors) and its magnitude (eigenvalues) (Balmith and Soliman, 2017). The eigenvectors, also called PCs, give the direction of the coordinated motion of C-alpha atoms, and the eigenvalues represented the magnitude of the motion with the corresponding eigenvectors. ON the basis of the covariance matrix C_ij_ for coordinates i and j, the principal elements of the protein motion were computed as the eigenvectors and defined by the following ensemble formula:

$$C_{ij}=<\left(X_{i}-<X_{i}>\right)\left(X_{j}-<X_{j}>\right)>,$$

where X_i/j_ are Cartesian atomic coordinates of the C-alpha atom i or j and <X_i_> and <X_j_> stood for the average coordinates derived from the MD simulation trajectory. The ProDy and VMD software were used to generate the PCA porcupine plot (Bakan et al., 2014).

### Cell–Cell Fusion Assay

The Spike protein, hACE2, and TMPRSSR2 proteins were purchased from MiaoLing Plasmid Sharing Platform (Wuhan, China). The V367F and Q498A mutants were constructed using overlapping PCR and cloned into a pcDNA3.1 vector. The primers used are: pc3.1NheI-f gga​gac​cca​agc​tgg​cta​gc; PC3.1XbaI-r ggg​ttt​aaa​cgg​gcc​ctc​tag​a; V367F-F gac​tac​tct​ttc​ctg​tac​aac​agc​gcc​tct; V367F-R tgt​aca​gga​aag​agt​agt​cgg​cca​cgc​a; Q498A-F acg​gct​tcg​cgc​cta​caa​acg​gcg​tgg​gc; Q498A-R ttg​tag​gcg​cga​agc​cgt​aag​act​gga​g. All the plasmids were sequenced after PCR.

The cell–cell fusion assay was conducted as previously reported (Yi et al., 2020). In brief, HEK293T cells were collected when they were ∼90% confluent in 10-cm dishes and then seeded into 24-well plates. The next day, pcDNA3.1-S/mutant S and pcDNA3.1-luc-RE (the plasmid was reconstructed on the pCDNA3.1 backbone and the renilla luciferase-coding region is under the control of the T7 promoter) were cotransfected and transferred in another well for subsequent cotransfection of pcDNA3.1-ACE2, pcDNA3.1-TMPRSSR2 and pCAG-T7pol (addgene# #59926). After 4–6 h of culture at 37°C with 5% CO_2_, the culture media were changed to DMEM (10% FBS), and the cells remained in culture for another 48 h. After 48 h of cotransfection, the two groups of HEK293T cells (Spike/mutant Spike and ACE2) were trypsinized and mixed at a 1:1 ratio and then plated on 96-well plates. The cells were further incubated at 37°C for 48 h, lysed with lysis buffer, and tested for luciferase activity (Promega, United States). The binding ability of mutants to ACE2 was analyzed by comparing the luciferase values (N = 3), the binding ability to ACE2 of mutants were analyzed by One-way ANOVA and Sidak’s multiple comparisons test (Software: GraphPad PRISM 6), *p < 0.05; **p < 0.01.

## Data Availability

The original contributions presented in the study are included in the article/Supplementary Material, further inquiries can be directed to the corresponding authors.
