# Supplementary figures and images for: Non-RBM Mutations Impaired SARS-CoV-2 Spike Protein Regulated to the ACE2 Receptor Based on Molecular Dynamic Simulation
Source: Front Mol Biosci. 2021 Jul 27;8:614443. doi: 10.3389/fmolb.2021.614443 (PMC8353372; doi:10.3389/fmolb.2021.614443)

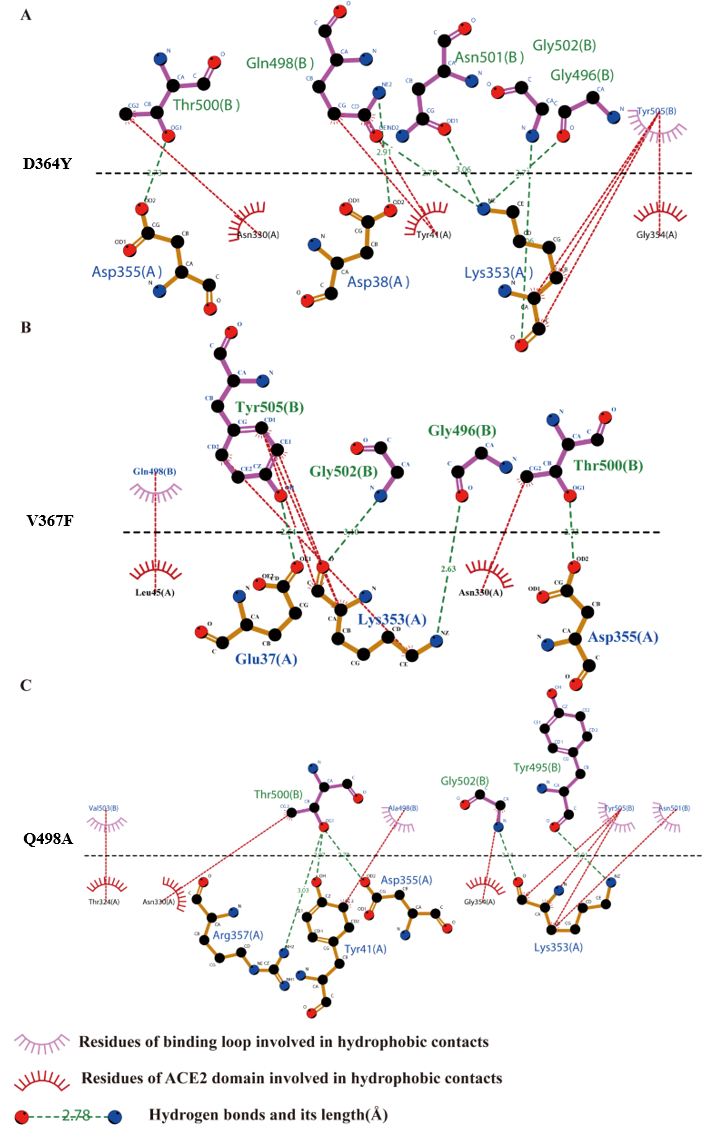

Supplement: Supplementary file 3 [file Image2.TIF]

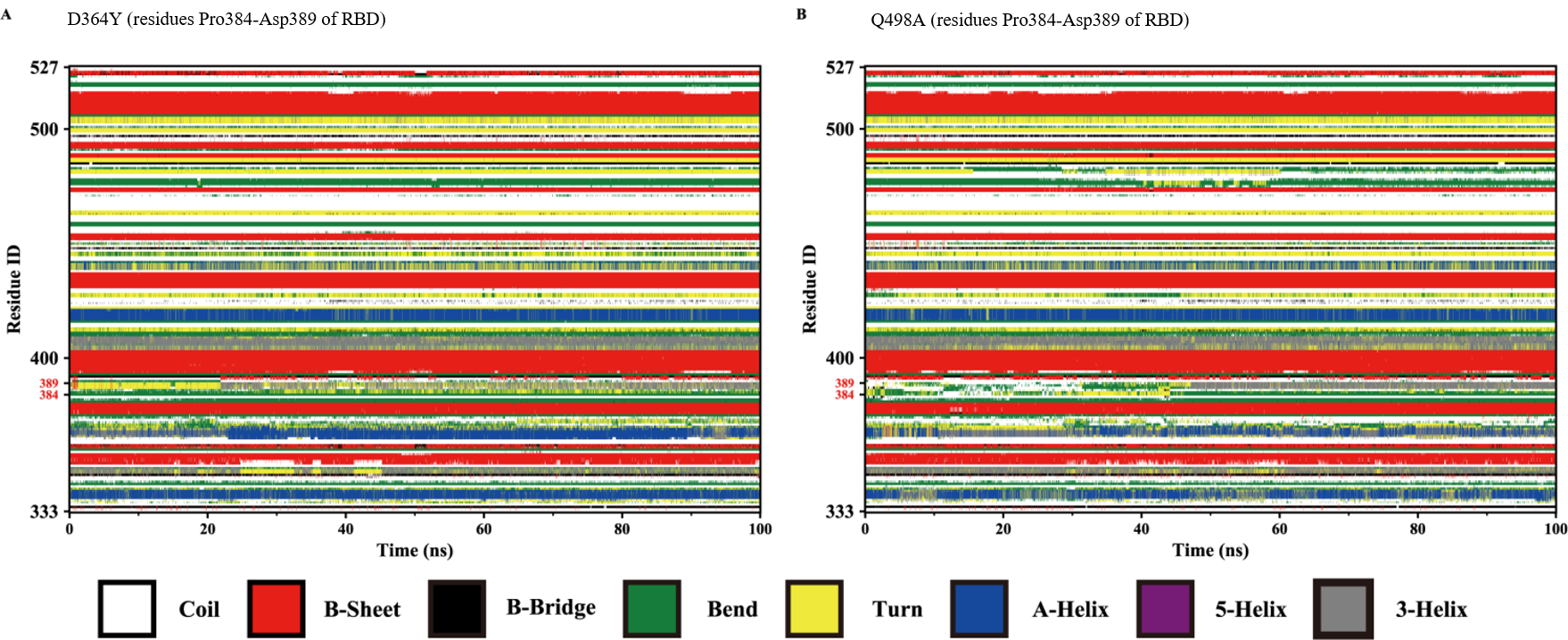

Supplement: Supplementary file 4 [file Image1.TIF]
